# Air pollution from forest burning as environmental risk for millions of inhabitants of the Brazilian Amazon: an exposure indicator for human health

**DOI:** 10.1590/0102-311XEN131422

**Published:** 2023-07-28

**Authors:** Igor Neves de Oliveira, Beatriz Fátima Alves de Oliveira, Ismael Henrique da Silveira, Lúbia Maieles Gomes Machado, Juliana Wotzasek Rulli Villardi, Eliane Ignotti

**Affiliations:** 1 Programa de Pós-graduação em Ciências Ambientais, Universidade do Estado de Mato Grosso, Cáceres, Brasil.; 2 Fundação Oswaldo Cruz, Teresina, Brasil.; 3 Instituto de Saúde Coletiva, Universidade Federal da Bahia, Salvador, Brasil.; 4 Programa de Pós-graduação em Saúde Coletiva, Universidade Federal de Mato Grosso, Cuiabá, Brasil.; 5 Vice-presidência de Ambiente, Atenção e Promoção da Saúde, Fundação Oswaldo Cruz, Rio de Janeiro, Brasil.; 6 Instituto de Estudos Avançados, Universidade de São Paulo, São Paulo, Brasil.

**Keywords:** Fires, Particulate Matter, Air Pollution, Conservation of Natural Resources, Incêndios, Material Particulado, Poluição do Ar, Conservação dos Recursos Naturais, Incendios, Material Particulado, Contaminación del Aire, Conservación dos Recursos Naturales

## Abstract

In this study, we propose an indicator of air pollution exposure to identify potential hazardous areas for human health in the Amazon and Central-West Regions of Brazil from 2010 to 2019. This indicator aggregates both concentrations and time of exposure to fine particulate matter (PM_2.5_), according to the current limit recommended by the World Health Organization (WHO). We used daily PM_2.5_ averages obtained from the Brazilian Health Integrated Environmental Information System (SISAM) to calculate the percentages of days with PM_2.5_ concentrations exceeding the limit of 15µg/m³ per year and per month. From 2010 to 2019, the months from August to October presented the largest areas and the highest percentages of days with unacceptable pollution concentration values, harmful to human health. These areas were concentrated in the Arc of Deforestation. Therefore, 60% of the residents of the Amazon and Central-West regions were subjected to inadequate air quality for approximately six months per year. The proposed indicator is reproducible and appropriate to monitor areas of exposure and risk for human health.

## Resumo

Este estudo propõe um indicador de exposição à poluição do ar para identificar potenciais áreas de risco para a saúde humana na região amazônica e no Centro-oeste do Brasil de 2010 a 2019. Esse indicador agrega as concentrações e o tempo de exposição à partículas finas de poluição (PM_2.5_), de acordo com o limite atual recomendado pela Organização Mundial da Saúde (OMS). Foram utilizadas médias diárias de PM_2.5_ obtidas do Sistema de Informações Ambientais Integrado a Saúde (SISAM) para o cálculo dos percentuais de dias cujas concentrações ultrapassaram o limite de 15μg/m³ por ano e por mês. De 2010 a 2019, os meses de agosto a outubro apresentaram as maiores áreas e os maiores percentuais de dias com valores de concentração inaceitáveis para a saúde humana. Tais áreas estavam concentradas na região do arco do desmatamento. Além disso, 60% dos moradores da região amazônica e do Centro-oeste eram expostos a uma qualidade inadequada do ar por aproximadamente seis meses por ano. O indicador proposto é reprodutível e adequado para monitorizar as áreas de exposição e de risco para a saúde humana.

## Resumen

Este estudio propone un indicador de exposición a la contaminación del aire para identificar posibles áreas de riesgo para la salud humana en la región amazónica y el Medio Oeste de Brasil de 2010 a 2019. Este indicador agrega las concentraciones y el tiempo de exposición a partículas finas de contaminación (PM_2.5_), de acuerdo con el límite actual recomendado por la Organización Mundial de la Salud (OMS). Se utilizaron los promedios diarios de PM_2.5_ obtenidos del Sistema Integrado de Información Ambiental en Salud (SISAM) para calcular el porcentaje de días cuyas concentraciones superaron el límite de 15μg/m³ por año y por mes. En la década de 2010 a 2019, los meses de agosto a octubre tuvieron las áreas más grandes y los porcentajes más altos de días con valores de concentración inaceptables para la salud humana. Tales áreas se concentraron en la región del arco de la deforestación. Además, el 60% de los residentes de la región amazónica y el Medio Oeste estuvieron sujetos a una calidad del aire inadecuada durante aproximadamente seis meses al año. El indicador propuesto es reproducible y adecuado para monitorizar las áreas de exposición y de riesgo para la salud humana.

## Introduction

Historically, Brazil has been constantly facing critical events of wildfires and burns. Over 36 years (1985-2020), Brazilian fires accounted for an area that is larger than England, burning about 150,957km² per year (1.8% of the country), which represents a cumulative number of 1,672,142km² (19.6%) consumed by burnings and/or wildfires. The most affected biomes, according to area proportion, were the Pantanal (57%), the Cerrado (36%), and the Amazon Forest (16.4%). The latter two represent 85% of the entire burned area ^1^.

In the Central-West and North regions of Brazil, where fire outbreaks have been more frequent, the upward movement of pollution is caused by the generated heat, the lower density of formed particles, and the gases in the air. These effects, associated with air currents, cause pollution dispersion from the local level to the regional and even continental levels ^2^. Fine particulate matter (PM_2.5_) is the major pollutant emitted by wildfires smoke, and its negative effects on human health are well known ^3^ ^,^ ^4^, including the increased morbidity and mortality due to cardiovascular and respiratory diseases, which especially affects the more vulnerable population subgroups such as children and older adults ^5^ ^,^ ^6^ ^,^ ^7^ ^,^ ^8^.

Forest fires are influenced by both climate and human-driven land-use changes. Strengthening environmental policies to control the incidence of fire can improve air quality and reduce negative impacts on public health ^3^. In the Brazilian Amazon, the reduction of deforestation rates from 2001 to 2012 resulted in a 30% reduction of PM emissions in wildfires, preventing about 400 to 1,700 premature deaths of adults per year ^3^. However, the increasing rates of deforestation from 2014 to 2019 have caused increased fire count and degraded air quality, which resulted in 3,400 (3,300-3,550) additional deaths in 2019 due to increased PM emissions ^9^.

In Brazil, concerns about the effects of air pollution on human health are mainly directed to metropolitan areas with air quality monitoring, where exposures mostly derive from fossil fuel or industrial production sources ^10^ ^,^ ^11^. The North Region - where most of the Brazilian Amazon is located - and the Central-West Region are less industrialized than other regions of Brazil; however, they present a large territory and 40 million inhabitants ^12^. During periods of increased burns, these inhabitants inhale polluted air up to five times higher than the maximum limits recommended by the World Health Organization (WHO) for PM_2.5_ ^13^. This population has limited access to information related to air quality levels. The government, along with environmental and health regulatory agencies, should regularly inform people regarding air quality ^14^. In the few localities where such information is provided, it is usually limited to daily and annual averages. However, for seasonal exposures caused by biomass burning, such measures may not express the annual magnitude of the exposure to poor air quality to which those residents are exposed since they do not have evidence of the time of exposure.

In 2010, a methodological approach based on the percentage of annual hours of PM_2.5_ > 80µg/m³ was proposed as an indicator of air pollution exposure in the Brazilian Amazon ^15^. This indicator showed an association with occurrences of respiratory diseases in the Brazilian Amazon region, as well as the feasibility of using an estimated environmental database. However, the application of this indicator was limited due to the unavailability of environmental data. Recently, with the restructuring of the Brazilian Health Integrated Environmental Information System (SISAM) and the availability of PM_2.5_ data estimated by the Copernicus Atmosphere Monitoring Service (CAMS), a new indicator to deal with the effects of forest fire on human health is possible. Thus, we propose an indicator of air pollution exposure to identify potential hazardous areas for human health in the Amazon and Central-West regions of Brazil from 2010 to 2019. This indicator aggregates both concentrations and time of exposure to the pollutant fine particulate matter (PM_2.5_), according to the current limit of 15µg/m³, recommended by the WHO ^16^.

## Material and methods

### Study area

The study area included the entire Brazilian Amazon and some surroundings areas, encompassing the three biomes that were most degraded by fire in recent years: the Amazon, the Cerrado, and the Pantanal. The study included all municipalities located in the Federative Units (UF) of the North region (Acre, Amapá, Amazonas, Rondônia, Pará, Roraima, and Tocantins), of the Central-West region (Federal District, Goiás, Mato Grosso, and Mato Grosso do Sul), and the State of Maranhão. Despite not being located in the Brazilian Amazon territory, the cities of the state of Maranhão located east of the meridian 44º were also included because they are part of the Cerrado biome. A total of 1,134 municipalities were included, corresponding to an approximate area of 6 million km^2^, equivalent to 68% of the Brazilian territory, with more than 42 million inhabitants in 2021 ^12^ ^,^ ^17^.

### Data source

To assess air quality, daily PM_2.5_ concentrations were used. Data were obtained from the reanalysis models of the CAMS, from the European Centre for Medium-Range Weather Forecasts (ECMWF) (CAMS-Reanalysis 2010-2017 and CAMS-Nrealtime 2018-2019), provided by Brazil’s SISAM. Data have a resolution of 0.125º and are provided for four times of the day: 12:00am, 06:00am, 12:00pm and 06:00pm, which were used to calculate the daily averages.

Hot spots refer to the points of fires or reference measured by satellites in accumulated quantities for each municipality. Data referring to burns and wildfires were obtained via total number of records of hot spots in the vegetation. These data are also available in the SISAM from 2002 onward.

### Calculation of the percentage indicator of days with inadequate air quality

According to the Global Air Quality Guidelines developed by the WHO ^16^, the daily average PM_2.5_ concentration has a limit of 15µg/m³. Based on this parameter, we calculated the percentages of days with PM_2.5_ concentrations exceeding the recommended limit, considered as “poor air quality” in our study. This indicator was calculated for all municipalities via the following equations - for each year of the studied series (a), for each month considering the entire decade (b), for the decade (c), and the month of each year (d):

$$\left(a\right)=\frac{\Sigma \;number\;of\;days\;in\;the\;year\;with{\;PM}_{2.5}>15\mu g/m^{3}}{365}*100\text{\%}$$

$$\left(b\right)=\frac{\Sigma \;number\;of\;days\;in\;the\;month\;\left(the\;decade\right)\;with\;{PM}_{2.5}>15\mu g/m^{3}}{10*30}*100\text{\%}$$

$$\left(c\right)=\frac{\Sigma \;number\;of\;days\;in\;the\;decade\;with{\;PM}_{2.5}>15\mu g/m^{3}}{365*10}*100\text{\%}$$

$$\left(d\right)=\frac{\Sigma \;number\;of\;days\;in\;the\;month\;with\;{PM}_{2.5}>15\mu g/m^{3}}{30}*100\text{\%}$$

### Data analysis

Maps of PM_2.5_ exposure indicators were created using the geostatistical interpolation method based on Inverse Distance Weighting (IDW), weighting by the inverse variance of distance of sample points in the study area. Thus, at the end of processing, it generates a file in raster format with 0.01º of distance to each grid point. The technique consists of predicting values for locations with no information by weighting the values of sample points, which, in this case, are represented by the indicator of each municipality. The method assumes that closer values will have a greater weight in the estimates than farther values ^18^. IDW interpolation is deterministic and establishes a known continuous surface ^19^. In this research, IDW interpolation maps were produced showing the annual and monthly percentages of “poor air quality”. Note that the metric error of the interpolation was not estimated. Deterministic interpolations are based directly on measured values close to sampled value and employ arbitrary or empirical models. No model error estimation was performed; thus, there is no strict assumption about the variability of a variable ^20^. Data and graphs were processed in the R programming language, version 4.0.2 (http://www.r-project.org), and the interpolation maps were made in ArcGIS, version 10.5 (http://www.esri.com/software/arcgis/index.html).

### Estimate of the population exposed to poor air quality

The total number of residents in the municipalities where percentage of days with poor air quality exceeded 25% per year was summed, equivalent to at least three months per year. Values on the population per municipalities were provided by the Brazilian Institute of Geography and Statistics (IBGE) and made available by the Brazilian Health Informatics Department (DATASUS). Details of the estimates are presented in the Supplementary Material (https://cadernos.ensp.fiocruz.br/static//arquivo/suppl-csp-1314-22_2784.pdf).

## Results

**Figure 1:**
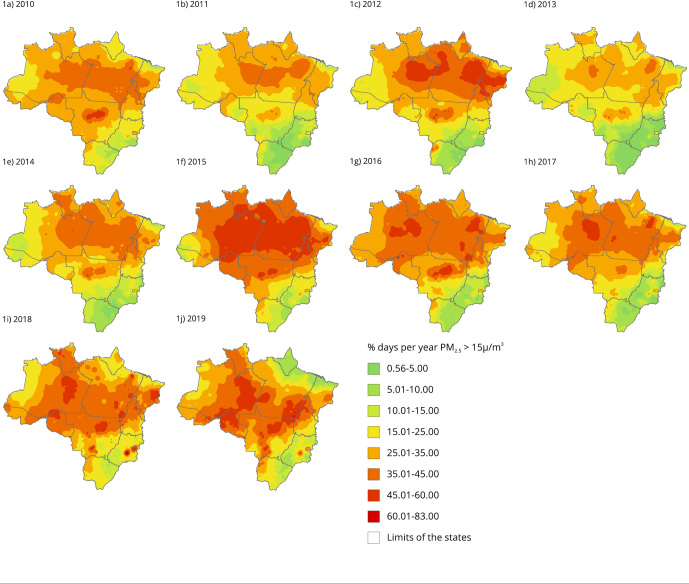
Annual percentage of days exceeding 15µg/m³ PM_2.5_ concentrations in the Amazon and Central-West regions of Brazil, 2010-2019.

The study shows large areas with more than 15% of days with poor air quality in all years. Thus, in two or more months of each year at least 20 million inhabitants were exposed to poor air quality (Figure 1 and Figure S1, Supplementary Material: https://cadernos.ensp.fiocruz.br/static//arquivo/suppl-csp-1314-22_2784.pdf). The largest areas occurred in 2015, with percentages ranging from 35% to 60%, which is equivalent to prolonged exposures of 4 to 6.5 months. The highest percentages were found in the states within the Brazilian Amazon.

**Figure 2:**
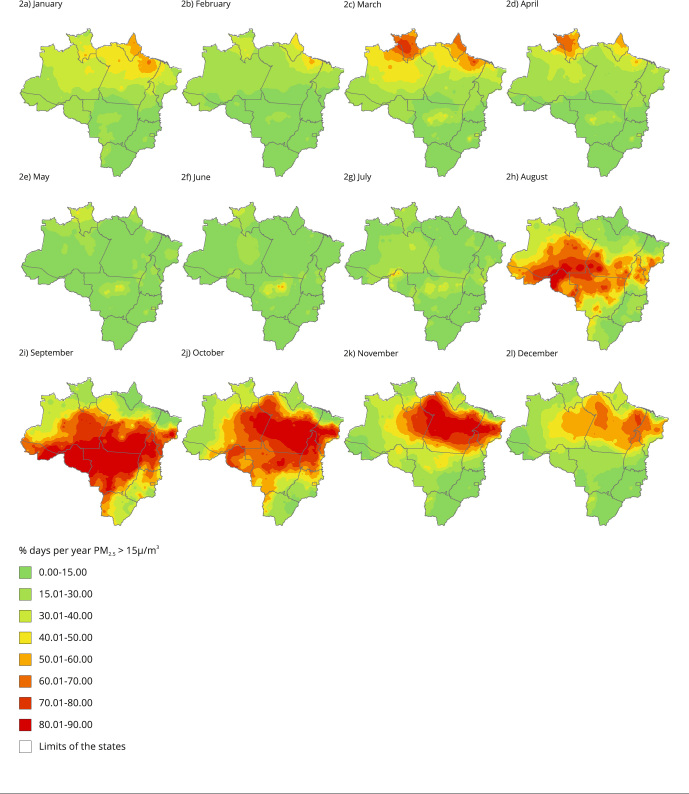
Monthly percentage of days exceeding 15µg/m³ PM_2.5_ concentrations in the Amazon and Central-West regions of Brazil, 2010-2019.

The monthly percentages show the seasonal pattern of exposure (Figure 2). The largest area can be seen from August to December, reaching 80% to 99% of the days of the month (25 to 30 days). The Brazilian Amazon extends from the western end of Acre to the south/southeast of Amazonas, center-south of Pará and Maranhão, on the border between the states of Tocantins and Goiás. It encompasses the entire states of Rondônia and Mato Grosso, reaching the Pantanal in the state of Mato Grosso do Sul. In the first four months of the year, percentages higher than 50% (more than 15 days/month), as well as those considered the highest percentages for the period, from 70% to 80% (20 to 25 days/month), are distributed only in northern Brazil, covering part of the states of Roraima and Amapá and some areas in the north of Amazonas and Pará - above the Equator line. The maps of hot spots complement the analysis about the areas presenting high levels of PM_2.5_, by showing the regions more frequently burned in the Brazilian Amazon and its surroundings (Figure S2 and Figure S3, https://cadernos.ensp.fiocruz.br/static//arquivo/suppl-csp-1314-22_2784.pdf).

In the Supplementary Material, Table S1 characterizes the time of exposure to “poor air quality” in all studied capitals and ten municipalities (all located in the central region of the Amazon) that presented the highest percentages of days with PM_2.5_ above of 15µg/m³. Table S2 complements this information by presenting the descriptive analysis for the capitals and the 10 municipalities (Supplementary Material: https://cadernos.ensp.fiocruz.br/static//arquivo/suppl-csp-1314-22_2784.pdf).

**Figure 3:**
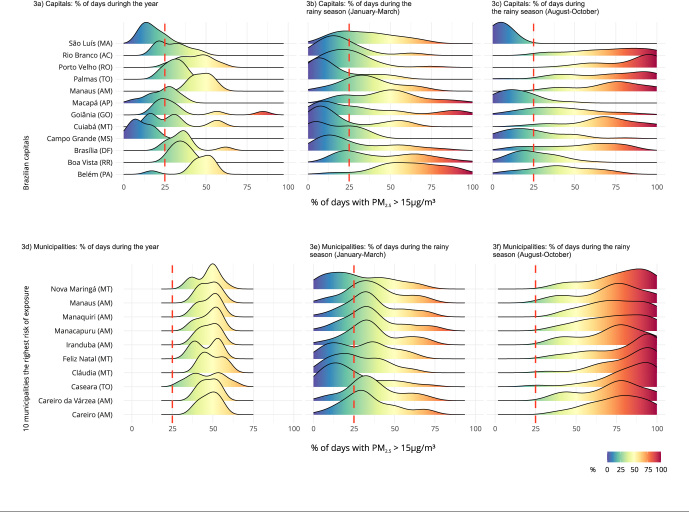
Distribution of the percentage of days with PM_2.5_ > 15µg/m³ in the capitals and municipalities with the highest risk of exposure in the Amazon and Central-West regions, according to the annual period and for the dry and rainy seasons.

AC: Acre State; AM: Amazonas State; AP: Amapá State; DF: Distrito Federal; GO: Goiás State; MA: Maranhão State; MS: Mato Grosso do Sul State; MT: Mato Grosso State; PA: Pará State; RO: Rondônia State; RR: Roraima State; TO: Tocantins State.

Figure 3 complements the description of the municipalities, as well as the state capitals, showing how the annual and quarterly percentages of high exposures identified in Figure 2 (from January to March and from August to October) are distributed. The period from August to October presented a longer exposure time to poor air quality, with 75% to 100% of the days in the 10 municipalities presented. The months from January to March influenced the exposure time in six municipalities in Amazonas, with percentages around 35%, approximately 30/90 days. For Boa Vista (Roraima State), Belém (Pará State), Macapá (Amapá State), and São Luís (Maranhão State), the estimated pollution levels for the first months of the year contribute to the percentage of days with poor air quality. For the other regions, the estimates for the months from August to October were used.

For the total population of the study area, at least 24 million inhabitants were exposed to poor air quality for at least three months per year, especially in the Amazon region, where more than half of its population (≅15 million) was subjected to this period of exposure every year. Annual estimates of the exposed population contingent, separately for the Amazon and Central-West regions, are presented in Figure S3a and Figures S3b (Supplementary Material: https://cadernos.ensp.fiocruz.br/static//arquivo/suppl-csp-1314-22_2784.pdf).

## Discussion

The percentage indicator of days in which PM_2.5_ concentrations exceeded the limits proposed by the WHO made it possible to combine concentration and time of exposure to characterize exposure to poor air quality, identifying areas of risk for human health. The indicator showed that millions of residents in the Brazilian Amazon and its surrounding areas were exposed to poor air quality for long periods every year due to wildfires and burns. The impacts of this exposure on human health have been clearly explained for decades ^16^. Specifically in the Brazilian Amazon, a series of studies show the harmful effects to health caused by pollution from biomass burning ^3^ ^,^ ^4^ ^,^ ^5^ ^,^ ^6^ ^,^ ^7^ ^,^ ^21^.

Despite not being included in the areas with the most critical records of hot spots, six of the ten municipalities with the highest percentages of days with poor air quality during 2010-2019 are in the central portion of the State of Amazonas. It is a region situated in the area of influence of the PM_2.5_ dispersion flow, from East to West in the dry season ^2^, and this is why some areas not involved in burnings are strongly affected by poor air quality ^15^.

The reduction in fires associated exclusively with deforestation can cause mean surface particulate matter concentrations to decline by approximately 30%, mainly in the dry season ^3^. However, the increased number of hot spots in the central portion of the Amazon, known as the Arc of Deforestation, shows the expansion of the deforested and burnt area in the region. The occurrence of hot spots in the Amazon and the practice of deforestation are closely related, even in years of milder droughts, and the main source of ignition is human action ^22^. The dynamics of hot spots, particularly in 2019, also reinforce the recent trend of increased forest burnings and deforestation associated with illegal occupation and land grabbing in this region ^23^.

The increasing deforestation of the Amazon biome and its surroundings in recent years ^24^, mainly the expansion of the Arc of Deforestation revealed by the Socio-environmental Institute ^25^, may cause this region to accumulate more combustible material, leading to increasingly severe fire seasons during the dry season ^26^. Consequently, we estimate the occurrence of long periods with poor air quality, as our results indicate municipalities with daily PM_2.5_ concentrations much above the acceptable level, with averages higher than 1,000µg/m³. The research highlights that the city of São Paulo, located in the southeast region of the country, presents around 80% of days with PM_2.5_ averages above the acceptable limit of 15µg/m^3^ from June to September, that is, between the winter and the beginning of spring, when rains are less frequent and temperature inversions occur. Even so, the maximum daily PM_2.5_ concentrations have not exceeded 70µg/m^3^ in the monitoring stations of the metropolitan area ^27^. In the Brazilian Amazon, air pollutant emissions generated by fires can expose children and adolescents to a PM_2.5_ concentration that is twice as high as the safe limit, which adverse health effects are not observed ^28^.

Studies conducted in municipalities of the Amazon and Central-West regions have reported the association between acute effects (immediate, lagged, and cumulative) on respiratory health, mainly in children and older adults, and exposure to increased PM_2.5_ concentrations (gradients of 3.5µg/m^3^, 5μg/m^3^ and 10µg/m^3^) ^4^ ^,^ ^5^ ^,^ ^6^ ^,^ ^7^ ^,^ ^29^ ^,^ ^30^ ^,^ ^31^ ^,^ ^32^ ^,^ ^33^ ^,^ ^34^ ^,^ ^35^.

Other studies have shown the relationship between these particulates and increased risks for development of chronic problems. Silva et al. ^36^ identified associations between exposure to high PM_2.5_ concentrations and low-weight newborns in municipalities located in the Amazon and the Cerrado. Alves et al. ^21^ found that exposure of human lung cells to inhalable particles in the Amazon significantly increases DNA damage and cell death, elucidating lung cancer development mechanisms mediated by aerosols from burns and wildfires.

The social problems faced by the regions increase the populations’ vulnerability to this environmental problem, as the most affected areas show few high-complexity hospitals and primary care services, which hinders access, mainly in the western portion of the Amazon, in the Arc of Deforestation (in southeastern Pará and northern Mato Grosso), and throughout the Pantanal biome ^37^.

Few studies conducted in Brazil have used indicators that combine level and time of exposure to investigate the association between air pollution and health outcomes in the Amazon. The annual percentage of hours with critical PM_2.5_ concentrations (> 80µg/m³), used by Ignotti et al. ^15^ and Silva et al. ^38^, as well as the annual percentage of hours with PM_2.5_ concentrations above 25µg/m³, used by Nunes et al. ^8^, were associated with hospitalizations due to respiratory and cardiovascular problems in susceptible groups. These findings suggest the possibility of using the indicator in epidemiological studies, in analyses of the health situation for environmental health surveillance, or, at least, in risk communications to the population and decision-makers.

According to Urrutia-Pereira et al. ^39^, despite the health impact caused by biomass burning in the Amazon Forest, the region still lacks public policies to improve the actions of health professionals and the transmission of information, aiming at protecting the population and improving the quality of life. We highlight that, today, few states have an air quality monitoring network based on low-cost PurpleAir PA-II-SP sensors (https://www2.purpleair.com/), and only in Acre State it is supported by the Public Prosecutor’s Office and by universities, among other collaborators (http://www.acrequalidadedoar.info). Other states, such as Mato Grosso State and Pará State, whose populations are extremely exposed and affected by the emission of air pollutants, do not have air quality monitoring systems.

In regions lacking monitoring stations, the use of modeled data can be a valid alternative. However, the lack of measured or observed data prevents the validation and/or application of correction factors to estimated exposure data, which is the main limitation of our study. Thus, the relevance of modeled data is undeniable, given the territorial and population scope of the estimates and their strategic use in the creation of public health policies. We emphasize the importance of ensuring that the 99th percentile of the annual distribution of average daily PM_2.5_ concentrations for 24 hours, as standardized by the WHO ^16^, must not exceed 3 to 4 days over the 15µg/m^3^ limit per year. In this study, we evaluated the frequency that this value is exceeded. For this reason, the excluding data above the 99th percentile does not result in differences in the percentage of days with exceeding 15µg/m^3^ PM_2.5_ concentrations . In other words, the period of exposure to high levels of PM_2.5_ is continuous and reaches, at least, three months every year in most regions.

The proposed indicator is reproducible and appropriate to monitor areas of exposure and risk for human health. The research shows that half of the Brazilian Amazon and Central-West Region population was exposed annually and for long periods to inadequate daily PM_2.5_ concentrations. We recommend the use of this indicator in epidemiological studies to investigate its relationship to health outcomes. Its application in environmental health surveillance systems and in the elaboration of effective monitoring policies/plans for the control of burns and wildfires is also important.
